# Simulating and Summarizing Sources of Gene Tree Incongruence

**DOI:** 10.1093/gbe/evw065

**Published:** 2016-03-26

**Authors:** Michael D. Woodhams, Peter. J. Lockhart, Barbara R. Holland

**Affiliations:** ^1^Discipline of Mathematics, School of Physical Sciences, University of Tasmania, Hobart, Australia; ^2^Institute of Fundamental Sciences, Massey University, Palmerston North, New Zealand

**Keywords:** phylogenetic tree, simulator, hybridization, coalescence, incongruence, gene trees

## Abstract

We introduce a gene tree simulator that is designed for use in conjunction with approximate Bayesian computation approaches. We show that it can be used to determine the relative importance of hybrid speciation and introgression compared with incomplete lineage sorting (ILS) in producing patterns of incongruence across gene trees. Important features of the new simulator are (1) a choice of models to capture the decreasing probability of successful hybrid species formation or introgression as a function of genetic distance between potential parent species; (2) the ability for hybrid speciation to result in asymmetrical contributions of genetic material from each parent species; (3) the ability to vary the rates of hybrid speciation, introgression, and divergence speciation in different epochs; and (4) incorporation of the coalescent, so that patterns of incongruence due to ILS can be compared with those due to hybrid evolution. Given a set of gene trees generated by the simulator, we calculate a set of statistics, each measuring in a different way the discordance between the gene trees. We show that these statistics can be used to differentiate whether the gene tree discordance was largely due to hybridization, or only due to lineage sorting.

## Introduction

Hybrid speciation and introgression are increasingly acknowledged as important biological processes (Mallet 2007; Mavarez and Linares 2008; Dunn et al. 2012; Abbott et al. 2013; Becker et al. 2013; Muhlfeld et al. 2014). Even estimates made more than 10 years ago suggested that at least 25% of plant species and 10% of animal species can hybridize with at least one other species (Mallet 2005). One consequence of hybrid speciation and introgression is that different loci within a genome may have different evolutionary histories. Depending on the relative rates of processes such as speciation, mutation, lineage sorting, and hybridization there will be different expectations regarding the level and kind of incongruence between different loci. The increase in power of sequencing technology means that it has become common to have datasets which include multiple genomic regions. By comparing patterns of incongruence in multi-gene datasets to patterns predicted under different scenarios we can hopefully gain a better understanding of the relative importance of reticulate evolution across a range of taxonomic groups.

Several multi-gene studies have found extensive incongruence between gene trees. Salichos and Rokas (2013) found no gene trees in common among 1,070 orthologous genes for 23 species of yeast. Cranston et al. (2009) found substantial levels of incongruence for 307 orthologous genes in six rice species, with only eight gene trees agreeing with the tree derived from a concatenated analysis. How to use this type of information to infer the evolutionary importance of reticulate evolutionary processes is still a developing area. Causes of incongruence are various and can be separated into different fundamental kinds. Gene trees can differ from each other for statistical reasons. Phylogenetic inference is error prone due to both stochastic error (noise) and systematic error (bias introduced by model misspecification). There are also biological reasons why gene trees might differ. This is the case even without invoking reticulate evolution, for instance, incomplete lineage sorting (ILS) (Maddison 1997), or gene duplication and subsequent loss leading to incorrect inference of orthology (Page and Charleston 1997). Finally, evolution of the species under consideration may have been a reticulate rather than strictly tree-like process; reticulate processes include horizontal gene transfer, hybrid speciation, and introgression.

A range of phylogenetic methods have been developed to deal with hybridization (Nakhleh 2011). Combinatorial approaches (e.g., Baroni et al. 2006; van Iersel and Kelk 2011) tend to ignore other possible sources of incongruence and focus on finding networks that display all the input information. Other approaches begin with character data and evaluate how well a particular network explains the data in either a parsimony or likelihood framework (Jin et al. 2006, 2007). The problem with this approach, noted by Jin et al. (2006), is that adding more reticulation to a network always explains character data better. Kubatko (2009) suggested using the Akaike information criterion (AIC) or BIC to decide what number of hybrid events should be preferred.

Other authors have focused on assessing whether hybridization has occurred in a particular data set. Holland et al. (2008) simulated data under the coalescent to see if super-networks had the power to distinguish hybridization from ILS. They used arbitrary networks, that is, hybridization was not explicitly modeled. Joly et al. (2009) used simulations on a species tree under the coalescent to establish a null distribution of minimum interspecies distances expected due to ILS; if, for a particular gene, an interspecies distance is smaller than predicted under an ILS only model then this was used to reject lineage sorting as an explanation for the data, and indicate hybridization.

More recently there have been approaches that attempt to model both ILS and hybridization and to compute exact likelihoods of different networks in small cases (Kubatko 2009; Meng and Kubatko 2009; Yu et al. 2011). Another approach, suggested in Holland (2013), is to include a hybridization rate and speciation rate as part of the model. Yu et al. (2012) took a similar approach to estimating relative rates of mutation and recombination in bacteria. If we abandon the aim of inferring exactly what network underlies the data in favor of trying to estimate the relative rates of different processes, this leads us to the importance of developing a flexible simulator of hybrid evolution.

There are several reasons why it would be useful to have a simulator of hybrid evolution that captured important biological aspects of reticulate evolutionary processes. First, it could be used to test how accurate new methods of species tree/network inference are in the presence of different levels and kinds of hybridization. Second, simulation is the foundation of Approximate Bayesian Computation (ABC), and the capacity to conduct realistic simulations is essential for ABC approaches used to test different hypotheses involving hybridization, for example, it might be used to compare different models of how species boundaries arise with genetic distance. To put it simply, we want the ability to simulate gene trees under a range of hybridization scenarios which can then be compared with patterns of gene tree incongruence in real datasets.

There are some current simulators of hybrid evolution, in particular Netgen (Morin and Moret 2006) specifically models hybrid speciation. Netgen is a forward-in-time simulator, but it only provides limited control over how genetic distance between pairs of species affects their chance of hybridization. Netgen is restricted to producing hybrids that are a 50/50 mix of the parental species. There are also population-based models of isolation with migration (Nielsen and Wakeley 2001; Hey and Nielsen 2007; Hey 2010). Isolation-with-migration (IM) models are focused on relatively recently diverged populations that are still undergoing gene flow and are designed to be used with population genetic data rather than sets of gene trees. IM models have been used within an ABC framework (Lopes et al. 2009; Sousa et al. 2012; Cornuet et al. 2014), in particular they have been used to successfully distinguish admixture from ancestral polymorphism (essentially equivalent to distinguishing introgression from ILS). However, with the IM simulators (Lopes et al. 2009; Cornuet et al. 2014) specific demographic scenarios need to be specified in advance, so there is not a straightforward way to use existing software to simulate under different relative rates of speciation and introgression (migration). These models do not directly consider hybrid speciation.

We wanted a simulator that would capture some of the main features that are thought to be important in describing hybrid evolution. In brief, we wanted to allow:

1. Flexible models of how the probability of hybridization changes as a function of genetic distance.
2. Asymmetry of inheritance of genetic material.
3. Different evolutionary time intervals (epochs) within which processes occur at different rates—such as periods of climatic instability where hybridization appears more common.
4. Incongruence of gene trees due to the coalescent process.

Regarding point 1, informed by the findings of genomes and genetic data, our understanding of the nature of species has been changing (Mallet 2007; Mavarez and Linares 2008; Dunn et al. 2012; Abbott et al. 2013; Becker et al. 2013; Lockhart et al. 2014; Muhlfeld et al. 2014), and has become a much more complex concept than the standard mathematical idealization where one branch splits instantaneously into two. While genetic incompatibilities between populations might build up gradually over time and space (Mallet 2005; Gourbiere and Mallet 2010; Abbott et al. 2013), ecological and morphological diversification can be rapid (e.g., Doebley 2006; Linder 2008; Lamichhaney et al. 2015). Studies on many eukaryotic organisms have shown that hybridization is common and frequent between species that are genetically close (Edmands 2002; Chapman and Burke 2007; Montanari et al. 2014). Several models have been put forward to explain how species boundaries arise. A simple model, where incompatibilities arise linearly through time, but have a multiplicative effect on fitness, results in the expectation that hybridization success should decline exponentially with genetic distance (Gourbiere and Mallet 2010). The Dobhansky–Muller model, where epistatic interactions are deemed to be most important, predicts a “snowball” effect where incompatibilities increase with the square of genetic distance (Gourbiere and Mallet 2010).

Regarding points 2 and 3, studies that have investigated divergence with gene flow and secondary contact between species suggest that across hybrid zones patterns of introgression can be asymmetric reflecting opportunities for backcrossing between hybrids and parental types (e.g., Coyner et al. 2015), genetic incompatibilities of mating types and also selection acting on phenotypic fitness. That is, the ability of a hybrid to compete with its progenitors will depend on environmental factors (Anderson and Stebbins 1954; Seehausen 2004; Arnold and Martin 2010) and these environmental factors might change through time. This complexity should be considered in simulations. Seehausen’s hybrid swarm hypothesis (Seehausen 2004), for example, might be modeled by assuming an epoch where hybridization is common followed by a period where hybridization is rare. Such a model could be extended further to consider Ehrendorfer’s Differentiation–Hybridisation Cycles (Ehrendorfer 1959) and Rattenbury’s cyclic hybridization driven by glaciation by allowing for alternating epochs where hybridization is more or frequent than in intervening epochs (Rattenbury 1962).

Regarding point 4, the coalescent process contributes a well-understood source of incongruence between gene trees that we think is important to include in the simulator.

Before continuing, we define some terminology. “Hybridization” is any instance of interbreeding between lineages which we consider to be separate species. This is used as a general term, used independently of the eventual outcome of the hybridization (e.g., gene flow or speciation). We only consider homoploid hybridization. “Hybrid speciation” is an event where hybridization founds a new species. Ordinary speciation we call “divergence speciation” when we feel the need to explicitly distinguish it from hybrid speciation. “Introgression” is a one way gene flow between species mediated by hybridization. That is, hybrids form, then breed back into one of the parent populations.

## Methods

### The Simulator

HybridSim is a forward-in-time event-based simulator. We use a forward-in-time approach so that we can make the probability of a hybrid establishing depend on genetic distance. This would not be possible in the traditional backwards-in-time coalescent framework, as at any particular step in the simulation, the program would not know the distance between the species at the tips. HybridSim models three processes: speciation (S), hybrid speciation (H), and introgression (I), which occur at rates that depend on the parameters *λ*_S_, *λ*_H_, and *λ*_I_, and *N*, where *N* is the current number of species. The three processes are memoryless, meaning that the time since the last event (be it speciation, hybrid speciation. or introgression) does not affect the rates at which the three processes occur.

Given that there are currently *N* species, nonhybrid (divergence) speciation occurs at rate $\lambda_{\text{S}}N$, that is, according to a Yule process (Yule 1925). Hybrid speciation involves a two-step process, hybrids are proposed at rate $\lambda_{\text{H}}N(N-1)$ with all pairs of leaves (taxa) equally likely. However, not all hybrid speciation events are successful. A proposed hybridization between taxon *a* and taxon *b* is successful with probability p=F(d) where *F* is some function of the genetic distance d:=d(a,b) between *a* and *b*. A successful hybrid speciation event results in a new hybrid species in addition to the two parents, which persist. The proportion of genetic material that the new hybrid species gets from each parent is determined by a user-defined discrete probability distribution. Introgression happens similarly to homoploid speciation with the difference that it does not create a new species; instead a portion of the genome of one species is overwritten by that portion of the genome from the other species. The probability of successful introgression is the same as for hybrid speciation, that is, p=F(d).

When an introgression event is successful, an arbitrary user-defined discrete probability distribution determines how much of the genome gets replaced—this distribution can be different than the one used for hybrid species formation.

The simulation begins with a speciation event giving two taxa separated by genetic distance of zero. The time to the next event is drawn from an exponential distribution with rate

$$\lambda =\lambda_{\text{S}}N+\lambda_{\text{H}}N(N-1)+\lambda_{\text{I}}N(N-1).$$

The type of the next event is then determined by drawing a uniform random number, *u*, and choosing speciation if $u<p_{\text{S}}$, an attempted hybrid speciation if $p_{\text{S}}\leq u<p_{\text{S}}+p_{\text{H}}$, and an attempted introgression otherwise, where

$$p_{\text{S}}=\lambda_{\text{S}}N/\lambda ;\;p_{\text{H}}=\lambda_{\text{H}}N(N-1)/\lambda ;\;p_{\text{I}}=\lambda_{\text{I}}N(N-1)/\lambda .$$

**Fig. 1.—:**
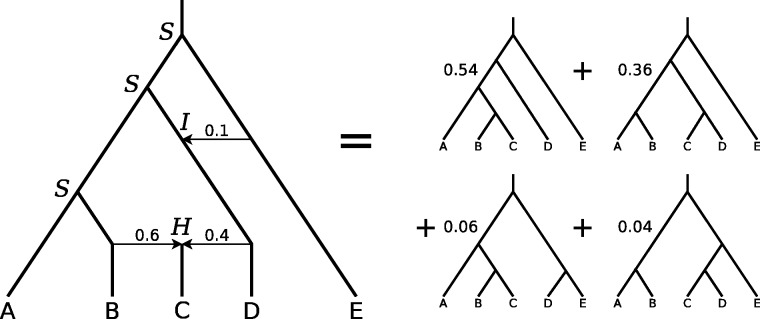
A hybrid species phylogeny with three divergence speciation events (marked *S*), one hybrid speciation (*H*) and one introgression (*I*). The resulting gene tree distribution is (((A,(B,C)),D),E); with weight 0.54, ((A,(B,C)),(D,E)); with weight 0.06, (((A,B),(C,D)),E); with weight 0.36, and ((A,B),((C,D),E)); with weight 0.04.

Figure 1 gives an example evolutionary scenario illustrating the three possible events and the resulting gene tree distribution.

A distance matrix is maintained throughout the simulation, and is updated after each event. For example, after a successful hybrid speciation, all off-diagonal entries are increased by twice the time interval leading up to the event, then a new row and column are created for the new taxon, with distances being a weighted average of those of its two parents. The distances used to determine hybrid speciation or introgression success chance are taken from this matrix.

We include five possible types of function that model the decreasing probability of successful hybridization as a function of genetic distance d:=d(a,b) between the proposed pair of taxa *a* and *b*. All of the functions require one user-specified parameter, *T*.

*Linear:* The probability of success declines linearly with genetic distance from 1 at *d* = 0 to a probability of 0 at some user-specified threshold *T*, beyond *T* the probability of successful hybrids forming is 0.

F(d)=max{0,1−d/T}

*Step function:* If the genetic distance is less than the threshold *T*, then hybridization will be successful, if it is greater than *T* then hybridization will fail.

$$F(d)=\{\begin{array}{ll} 1 & :d\leq T;\\ 0 & :\text{otherwise} \end{array}$$

*Quadratic:*

$$F(d)=\text{max}\{0,1-{\left(d/T\right)}^{2}\}$$

*Snowball:*

$$F(d)=e^{-d^{2}/T}$$

*Exponential decay:*

$$F(d)=e^{-d/T}$$

(If it is required that hybridization success be independent of distance, this can be achieved by using the step function, with threshold *T* set to at least twice the maximum tree depth.)

In summary, the main loop of the simulation is as follows.

1. The time to the next event and the type of that event (speciation by divergence, attempted hybrid speciation, or attempted introgression) are randomly selected.
2. Halting conditions are checked and the simulation may be halted.
3. The phylogeny and genetic distance matrix are updated to account for the passing of time until the next event.
4. If the event is speciation, a random leaf node is selected as the parent. The phylogeny and genetic distance matrix are updated so that a child species is created. Continue from step (1).
5. If the event is an attempted hybrid speciation or attempted introgression, two leaf nodes are chosen at random, and the genetic distance between them determined.
6. The reticulation success function is applied to this distance. If this results in failure, continue from step (1).
7. If the reticulation succeeds, update the phylogeny and distance matrix (increasing the number of leaves by one if it was a hybrid speciation event.) Continue from step (1).

Rates of the three processes need not remain fixed throughout the simulation, they can be different in each epoch. The form of the function that controls the decline in probability of hybrid success must be the same for each of the epochs, but the parameter, *T*, can change from one epoch to the next.

The simulation of the hybrid phylogeny stops when any one of three conditions is met: (1) the next event would cause the root to tip distance to exceed a user-specified maximum; (2) the next event would cause the number of species to exceed a user-specified maximum; or (3) the next event would cause the number of successful hybrid speciation or introgression events to exceed a user-specified maximum. In case (1), time is advanced to make the root-to-tip distance equal the specified maximum. In cases (2) and (3) time is advanced to the point where the limit-violating event would have occurred.

Optionally, we can specify a minimum number of reticulation events that must occur in the hybrid phylogeny. If a simulated phylogeny does not reach this minimum, it is discarded and a new phylogeny simulated. We can also specify a number of reticulation events to reduce to. If the phylogeny has more reticulation events than this, reticulations are eliminated (effectively setting the gene contribution from one of the parents to 100%) at random until this limit is reached. By setting these two limits to the same value, we can create a phylogeny with a specified number of reticulation events. (Note that eliminating reticulation events in this way can lead to inconsistency: one reticulation event may have been able to succeed only because an earlier reticulation event decreased the distance between the interacting taxa. We could eliminate the earlier event and keep the later event, resulting in a reticulation between taxa too distantly related. We recommend the reticulation reduction capability only be used when reticulation success is independent of distance between taxa.)

We implement a restricted version of the coalescent within a hybrid phylogeny. For each locus, we convert the hybrid network into a “lineage” tree by randomly choosing one of the two parents for each reticulate node (with probabilities given by the genetic contribution of each parent to that node). We then perform a coalescent simulation within the lineage tree to produce a gene tree for the locus, starting with a single lineage at each leaf. The coalescence rate, *λ*_C_, is a user-supplied parameter. For a branch of length *t*, with two uncoalesced lineages at its base, the probability the lineages will remain uncoalesced by the top of the branch is $p=e^{-t\lambda_{\text{C}}}$. (*λ*_C_ can also vary by epoch.)

The first type of output available is a probability distribution on a set of lineage trees, where each tree gets weighted according to the proportion of the genome that evolved on that tree. In cases where the number of reticulate events is large this might not be tractable, in this case the user can specify a number of trees to sample from this distribution without the probability distribution on trees ever explicitly being computed.

If it is desired to model sources of error that arise due to phylogenetic inference then sequence data will also be required. The simulation output gives edge-weighted trees in Nexus format and so can be easily pipelined to produce sequence data under a wide range of models of sequence evolution. We have also provided the option of simulating character data under the Dollo model as some recent sequencing technologies (DArT) produce data of this kind (Woodhams et al. 2013).

The HybridSim software is available at https://github.com/MichaelWoodhams/HybridSim, including a manual (with advice on suitable parameter settings) and sample input files.

### Base Statistics

ABC is an increasingly popular approach to statistical inference in cases where the likelihood function is intractable. It has widespread application in population genetics. Several recent review articles give an overview of the technique in the population genetics context (Beaumont 2010; Csillery et al. 2010). Very briefly, the idea is to repeat the following steps a large number of times. (1) Pick parameters for a model of interest according to a prior distribution (e.g., in our case these parameters might be the divergence speciation rate, introgression rate, hybrid speciation rate, and the parameter *T* that controls the probability of successful hybrid formation). (2) Simulate a dataset using the parameters. (3) Calculate base statistics on the simulated datasets. Optionally, these base statistics may be transformed in some way into a (usually smaller) set of summary statistics, which may have useful properties such as lacking collinearity or improved sensitivity to the parameters being estimated. (4) Once a large number of simulations have been performed, their summary statistics are compared with the summary statistics for the real data, and only the “closest” simulations are retained.

To reiterate, base statistics are calculated directly from the data, and are often statistics with a long history of use with that type of data. Summary statistics are the criterion for how closely a simulation matches the real data. It is possible to use base statistics directly as summary statistics, but more generally the summary statistics are some transformation (e.g., linear combination) of the base statistics.

For ABC to work effectively it is important to have summary statistics that capture useful information about the underlying processes. Ideally, you would like *sufficient* statistics (i.e., ones that are maximally informative about the parameters of interest) but this is often not possible (Joyce and Marjoram 2008; Fearnhead and Prangle 2012). In the population genetics setting people have used traditional statistics such as the number of unique haplotypes, the number of segregating sites, the average pairwise difference between sequences, Tajima’s *D*, and *F*_ST_ as their base statistics.

We want to measure levels and kinds of incongruence among gene trees. As computing summary statistics on trees is not a well-established field, we have compiled a set of base statistics, which we listed below. As the ABC approaches rely on doing large numbers of simulations, it is important that the base statistics be quick to calculate.

We first introduce some notation. Let $P=(T_{1},T_{2},\ldots ,T_{n})$ be a collection of phylogenetic trees, each on the same set of taxa, *X*. We refer to *P* as the *tree profile*. Let T(P) be the set of unique tree topologies in the tree profile. Let *w*(*T*) be the number of times tree topology *T* appears in the tree profile. Let $d_{\text{RF}}(T_{1},T_{2})$ be the Robinson–Foulds distance between trees *T*_1_ and *T*_2_.

A *split* $A|\bar{A}$ is a bipartition of the taxa set *X*, that is, $A\cup \bar{A}=X$, and $A\cap \bar{A}=\emptyset$. Two splits $A|\bar{A}$ and $B|\bar{B}$ are said to be *compatible* if at least one of the sets $A\cap B,A\cap \bar{B},\bar{A}\cap B$, or $\bar{A}\cap \bar{B}$ is empty, otherwise the splits are *incompatible*. A tree is said to *display* a split if there is an edge in the tree whose removal would decompose the tree into two components, one with leaf labels *A* and the other with leaf labels $\bar{A}$. Let *S*(*T*) be the set of splits displayed by *T*. Let $S=\cup_{i=1}^{n}S(T_{i})$ be the set of unique splits displayed by the trees. Let $w_{\text{S}}(s)$ be the number of trees that display split *s*.

A *quartet* is a 4-taxon tree. A tree is said to display a quartet q=ab|cd if the unrooted tree restricted to taxa *a*, *b*, *c*, and *d* is (*a*,*b*,(*c*,*d*)). Let $w_{Q}(q)$ be the number of trees that display quartet *q*.

A *cherry* is an internal node in a tree that has two incident pendant edges. Let *C*(*T*) be the set of cherries displayed by *T*. Let $C=\cup_{i=1}^{n}C(T_{i})$ be the set of unique cherries displayed by the trees.

We have implemented the following base statistics:

- Tree entropy (TE):
  $$\sum_{T_{i}\in T(P)}w(T_{i})\text{log}(w(T_{i}))$$
- Quartet entropy (QE):
  $$\sum_{a,b,c,d\in X}\sum_{i=1}^{3}w_{Q}(q_{i})\text{log}(w_{Q}(q_{i}))$$
  where $q_{1}=ab|cd,q_{2}=ac|bd$, and $q_{3}=ad|bc$.
- Split incompatibility (SI): This statistic can be expressed in two different forms, the first is more intuitive, the second is easier to generalize to a family of statistics
  $$\text{SI}=\sum_{T_{i},T_{j}\in P}d_{\text{RF}}(T_{i},T_{j}),\text{ or}$$
  $$\text{SI}=\sum_{s_{i},s_{j}\in S}w_{\text{S}}(s_{i})w_{\text{S}}(s_{j})I(s_{i},s_{j}),$$
  where $I(s_{i},s_{j})$ is an indicator function that evaluates to 1 if splits *s_i_* and *s_j_* are incompatible and 0 if they are compatible.
- Threshold *SI* (*SI*-*k*): Similar to *SI*, but it ignores splits with weight less than or equal to *k*.
  $$SI\text{-}k=\sum_{s_{i},s_{j}\in S\prime }(w_{\text{S}}(s_{i})-k)(w_{\text{S}}(s_{j})-k)I(s_{i},s_{j}),$$
  where S′={s∈S|w(s)>k}, and *I* is defined as above.
- Rare splits (RS): Counts the number of splits that only appear in a single tree
  $$\text{RS}=\left|\left\{s_{i}:w_{S}(s_{i})=1\right\}\right|$$
- Distance to consensus (DC):
  $$\text{DC}=\sum_{T_{i}\in P}d_{\text{RF}}(T_{i},M),$$
  where *M* is the majority-rule consensus tree *M* for the tree profile. We note that this is equivalent to
  $$\sum_{s\in S}\text{min}\{w_{S}(s),n-w_{S}(s)\},$$
  so the consensus tree does not actually have to be calculated.
- Unique cherries (UC): |C|. Number of unique cherries found across the trees
- Unique splits (US): |S|. Number of unique splits found across the trees
- Tree Certainty (TC): Salichos and Rokas (2013) define the “internode certainty” of a split as $1+p{\text{log}}_{2}p+(1-p){\text{log}}_{2}(1-p)$ where $p=\frac{x_{1}}{x_{1}+x_{2}}$ and *x*_1_ is the number of times the split is observed, *x*_2_ is the number of times the most numerous incompatible split is observed. Then Salichos et al. (2014) define TC of a tree as the sum of internode certainties over the splits in a tree. We use the greedy consensus tree (Bryant 2003) as the tree to evaluate the TC on.
- Tree Certainty All (TCA): Salichos et al. (2014) also define a generalization of internode certainty, “internode certainty all” (ICA) which considers multiple splits conflicting with the one being evaluated, and a corresponding “TCA” statistic summing ICA over a tree. Again we evaluate this on the greedy consensus tree. Salichos et al. (2014) suggest neglecting conflicting splits below some threshold, but we have not done this.

### Simulation Study

To test if the base statistics described above have any power to distinguish different causes of incongruence we use the new simulator to generate tree profiles under a range of scenarios. We fit multinomial regression models which use the base statistics (TE, SI, DC, UC, US, QE, RS1, SI-1, TC, TCA) and their interaction terms as explanatory variables in order to predict the simulation scenario. The models were fit using the multinom function from the nnet package in R (Venables and Ripley 2002). The full model was fit initially and then updated using the stepAIC function, this can remove terms that do not improve the AIC score of the model. The multinomial fit was performed on one set of simulations (250 simulations for each of four sets of parameters) and then cross-validated on a second set of simulations, differing only in random number generator seed from the first set. Results of the cross-validation classification are shown as confusion matrices. We acknowledge that although being able to successfully classify scenarios does not capture the full difficulty of an ABC inference procedure it is an important precursor to attempting to use such methods.

The simulation parameters are shown in table 1. We consider only two sources of gene tree incongruence—lineage sorting (deep coalescence) and reticulation. Example one is designed to be a fairly easy problem, where hybrid speciation is readily recognized. It includes four scenarios with two different levels of hybridization: zero or two hybrid speciation events; and two different levels of coalescence rate: fast (rate $15\lambda_{\text{S}}$) or slow (rate $5\lambda_{\text{S}}$). Slow coalescence increases the impact of lineage sorting. Example two is designed to be a harder problem. It consists of four scenarios, these range from no hybrid speciation ($\lambda_{\text{H}}=0$) and large lineage-sorting effects (coalescence rate = $6\lambda_{\text{S}}$) to high hybrid speciation and introgression rates ($\lambda_{\text{H}}=0.4$) and small lineage-sorting effects (coalescence rate = $1000\lambda_{\text{S}}$). The four scenarios were chosen such that the mean number of unique splits (*TS* statistic) varied little between scenarios. In contrast to example 1, parental contributions during reticulation are less even. Also in example 2 we impose a linear hybridization success function, so that reticulation events will be more local. Example 2 uses 150 gene trees. Example 3 is identical to example 2 except we have decreased the number of gene trees to 10, to make classification harder again.

**Table 1:** HybridSim Parameters for the Simulations.

	Example 1	Example 2	Example 3	Example 4
Number of gene trees	50	150	10	106
Number of Hybrid speciations	0 or 2	Variable		0–3
Hybrid and introgr. Rate	N/a	(0,0.1,0.26,0.4)		N/a
Hybridisation success	Always	Linear, threshold *T* = 1.3		Always
Coalescence rate	5 or 15	(6,10,30,1000)		2–200
Hybrid gene contribution	50:50	75:25		50:50
Introgression gene contribution	N/a	90:10		N/a
Number of taxa	10	20		8
Number of simulations	2,000	1,000		100,000

Note.—Speciation rate is 1 in all simulations, so all other rates are given relative to the speciation rate. Example 4 is an ABC analysis of the Rokas et al. (2003) data for eight yeast species.

### Approximate Bayesian Computation

Example 4 is an ABC on the Rokas et al. (2003) dataset of 106 genes from eight yeast species. We simulate 106 gene trees on eight taxa allowing only hybrid speciation events (no introgressions) in which each parent contributes 50% of the genome. The range of coalescence rates (2–200), hybrid speciation events (0–3), and number of simulations (100,000) were selected after preliminary analyses.

## Results

### Example 1

**Fig. 2.—:**
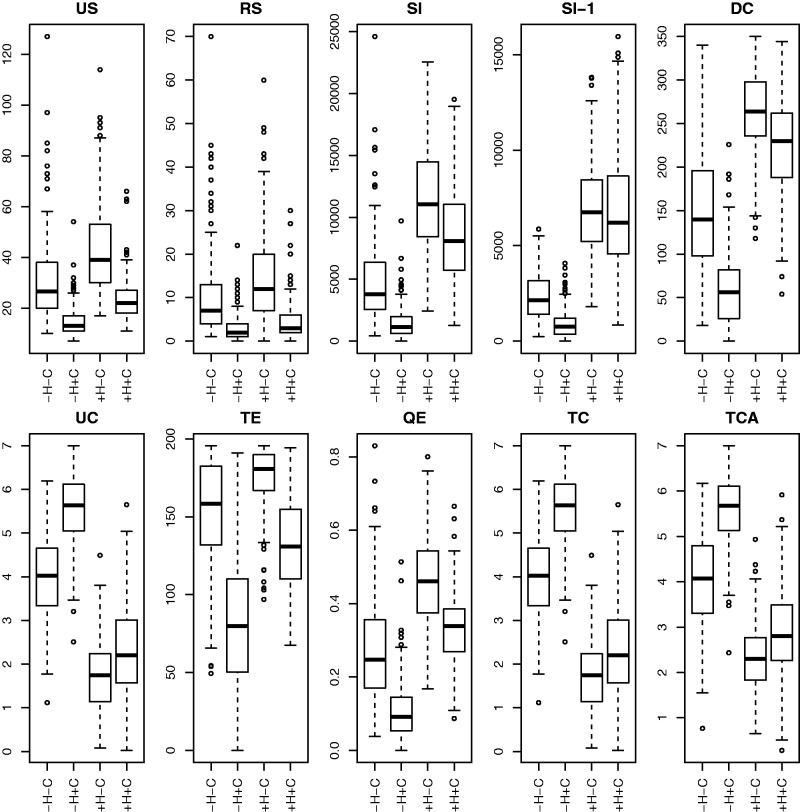
Boxplots of ten base statistics under the four different simulation scenarios from example 1. “−H” and “ +H” are 0 or 2 hybridizations, “−C” and “+C” are slow (rate = 5) or fast (rate = 15) coalescence.

**Fig. 3.—:**
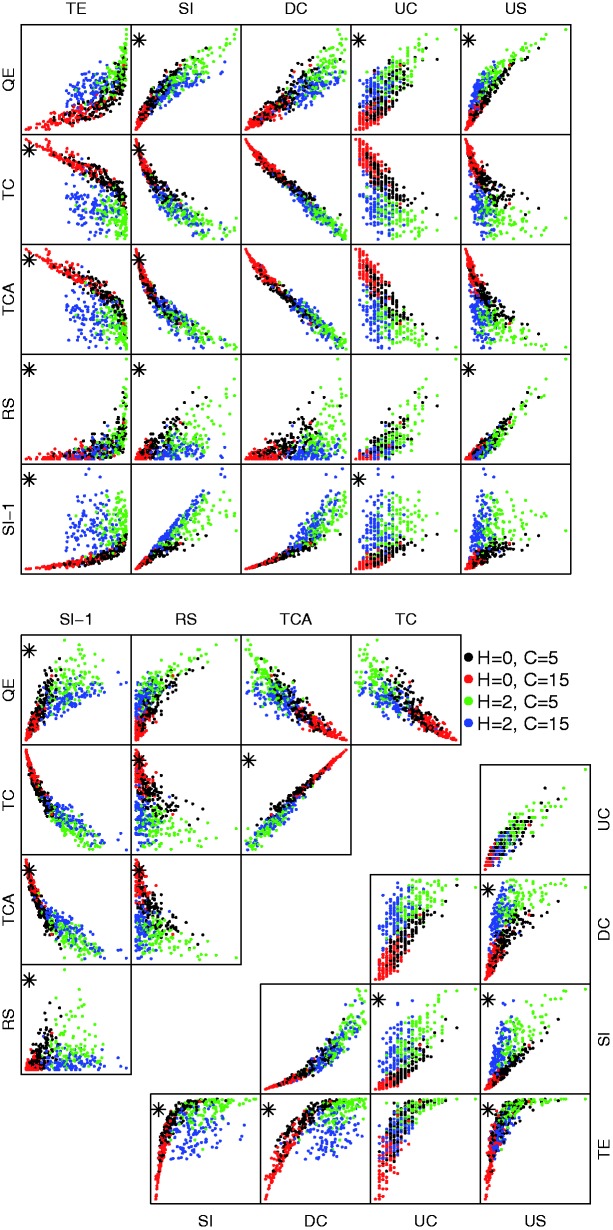
Pairs plots of base statistics for example 1. Asterisks indicate the two-way interaction terms which were included in the multinomial fit. A random 500 points of 2,000 are plotted to avoid overcrowding.

In example 1, we expect it to be fairly easy to correctly classify the scenarios. Both boxplots of individual base statistics, figure 2, and scatter plots of pairs of base statistics, figure 3*a*, suggest that they do have the power to distinguish between sets of gene trees generated under different scenarios.

After beginning with a model with all two-way interaction terms and then using the stepwise AIC procedure we ended up with a model with all ten main effects and 24 of the 45 possible interaction terms (indicated by a star in fig. 3*a*). Table 2 shows the confusion matrix for the best-fitting multinomial model. Overall 845 of the 1,000 sets of gene trees are correctly classified. Eight hundred and fifty-five have correct hybridization classification (but possibly wrong coalescence rate) and 989 have the correct coalescence rate classification (but possibly wrong hybrid speciation rate).

**Table 2:** Confusion Matrix for Four Scenarios in Example 1.

Actual condition	**Predicted condition**			
	**H0, C5**	**H2, C5**	**H0, C15**	**H2, C15**

H0, C5	214	35	0	1

H2, C5	30	219	0	1
H0, C15	6	0	204	40
H2, C15	0	3	39	208

Note.—Rows give the true classification and columns give the classification as predicted by the best-fit multinomial model with first-order interaction terms with ten base statistics (TE, SI, DC, UC, US, QE, TC, TCA, RS1, and SI-1). 1,000 simulations were used as training data to generate the multinomial fit, then a fresh 1,000 simulations were evaluated to generate this table.

### Example 2

**Fig. 4.—:**
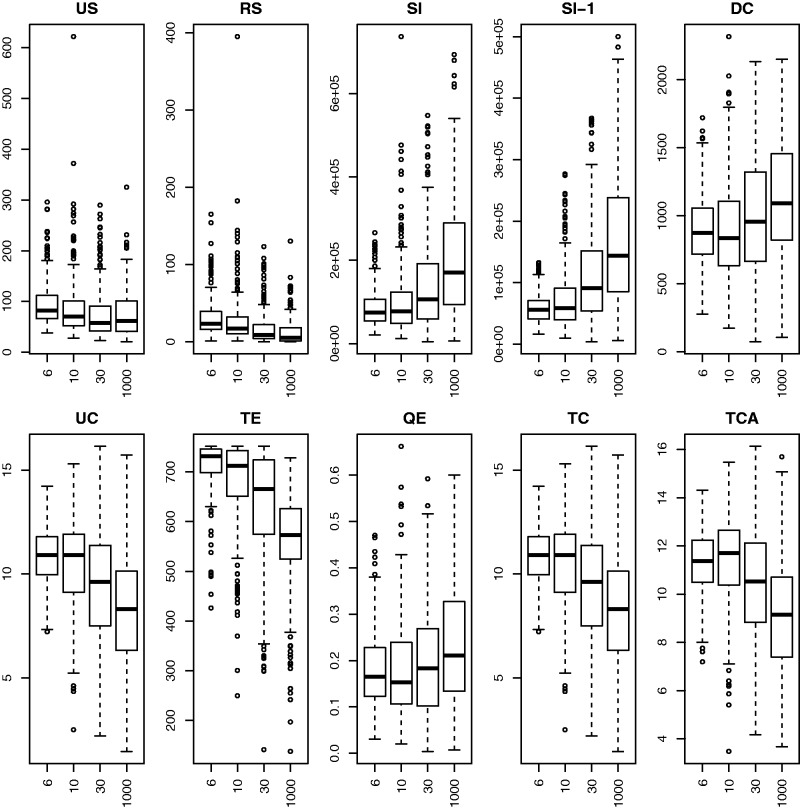
Boxplots of ten base statistics under the four different simulation scenarios from example 2.

**Fig. 5.—:**
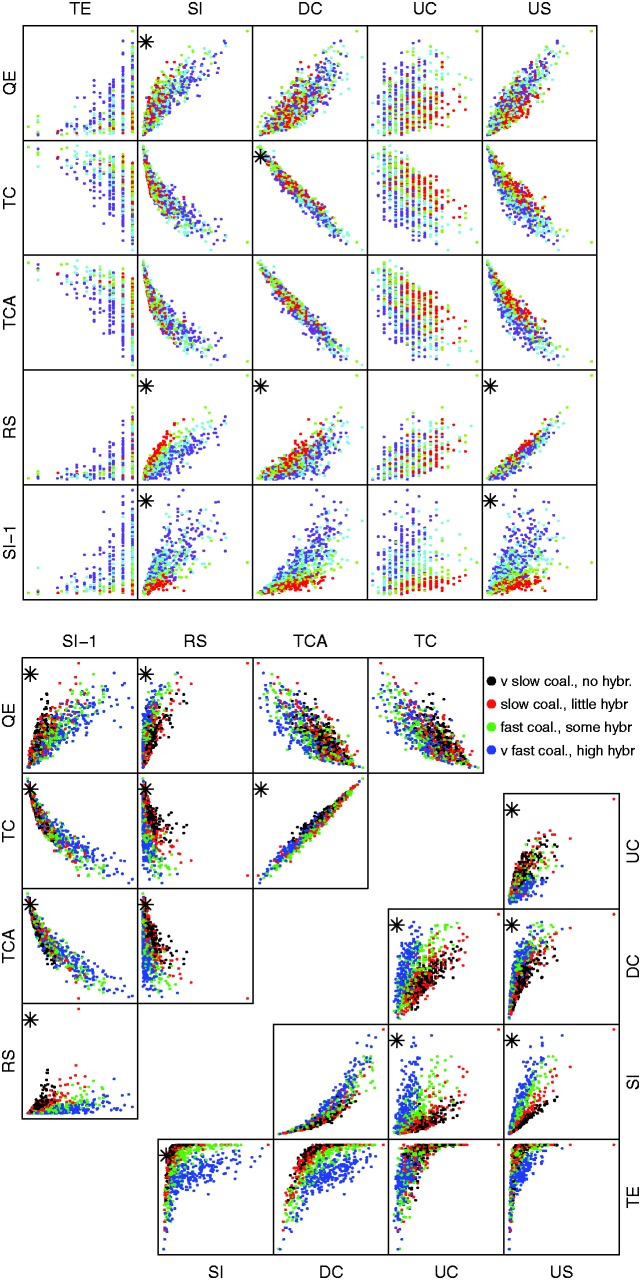
Pairs plots of base statistics for example 2. Asterisks indicate the two-way interaction terms which were included in the multinomial fit.

In example 2, we have ample data (150 gene trees) but mixtures of coalescence and hybrid speciation which may be difficult to distinguish. Boxplots of individual base statistics, figure 4, show that by forcing the scenarios to have roughly similar *US* statistics, several of the other statistics (*RS*, *SI*, *DC*, *QE*) look like they have little discriminatory power taken individually. The scatter-plots of pairs of base statistics, figure 5*a*, also suggest that it may be difficult to distinguish between sets of gene trees generated under different scenarios.

After beginning with a model with all two-way interaction terms and then using the stepwise AIC procedure we ended up with a model with all ten main effects and 36 of the 45 possible interaction terms. Table 3 shows the confusion matrix for the best-fitting multinomial model. Overall 836 of the 1,000 sets of gene trees are correctly classified in the cross validation. The misclassifications are usually to a nearby scenario.

**Table 3:** Confusion Matrix for Four Scenarios in Example 2 (150 Gene Trees), Using Cross-Validation (See Text).

	**C6**	**C10**	**C30**	**C1000**
C6	216	34	0	0
C10	45	178	26	1
C30	0	27	210	13
C1000	0	0	18	232

Note.—Rows give the true classification and columns give the classification as predicted by the best-fitting multinomial model.

The Supplementary Material contains Splitstree (Huson and Bryant 2006) consensus networks (Holland et al. 2004) for some random tree sets from this simulation. As expected, as the hybrid speciation rate increases, so in general does the nontreeness of the consensus network, but a given hybrid speciation rate can result in very different consensus network appearance.

### Example 3

**Fig. 6.—:**
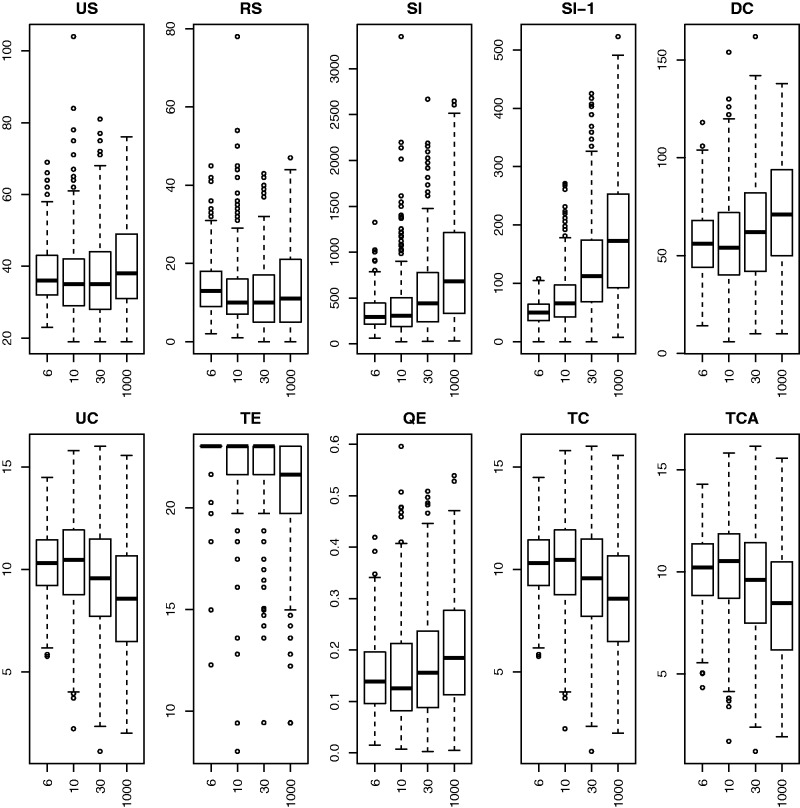
Boxplots of ten base statistics under the four different simulation scenarios from example 3.

In example 3, we have the same scenarios as example 2, but with only ten gene trees we expect classification to be difficult. The boxplots, figure 6, are similar to those of example 2.

**Fig. 7.—:**
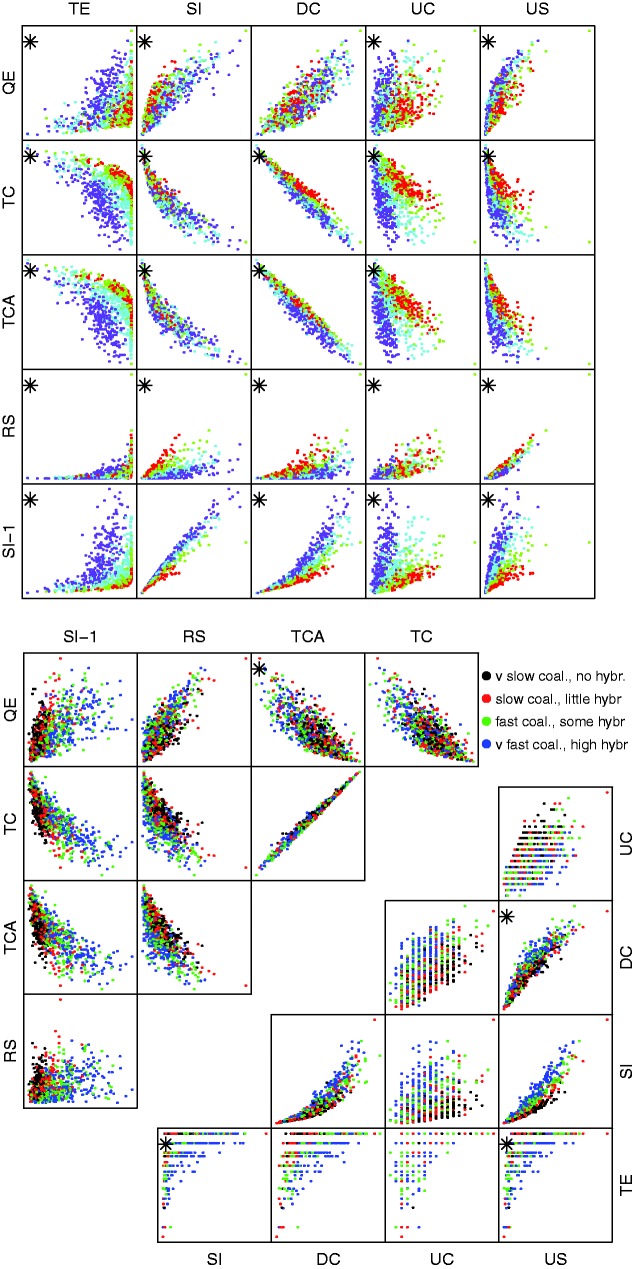
Pairs plots of base statistics for example 3. Asterisks indicate the two-way interaction terms which were included in the multinomial fit.

After beginning with a model with all two-way interaction terms and then using the stepwise AIC procedure we ended up with a model with all ten main effects and 11 of the 45 possible interaction terms (fig. 7*a*). Table 4 shows the confusion matrix for the best-fitting multinomial model. Overall 619 of the 1,000 sets of gene trees are correctly classified. The misclassifications are usually to a nearby scenario.

**Table 4:** Confusion Matrix for Four Scenarios in Example 3.

	**C6**	**C10**	**C30**	**C1000**
C6	187	59	4	0
C10	79	123	45	3
C30	7	61	134	48
C1000	1	9	65	175

Note.—Rows give the true classification and columns give the classification as predicted by the best-fitting multinomial model.

### Example 4 (ABC)

The simulation parameters, for example, four, match the Rokas et al. (2003) yeast dataset in number of gene trees (106) and taxa (8). The base statistics of the gene trees found by Rokas et al. (2003) are shown in table 5.

**Table 5:** **Base Statistics for the 106 Gene Trees of the** Rokas et al. (2003**) Eight Taxon Yeast Dataset.**

**TE**	**QE**	**SI**	**SI-1**	**SI-2**	**RS**	**DC**	**UC**	**US**	**TC**	**TCA**
254.5	0.3956	18151	12427	9952	9	276	9	26	2.101	2.613

Our simulations vary in two dimensions: the number of hybrid speciation events, and the coalescence rate. Fearnhead and Prangle (2012) recommend an approach to ABC which we follow here. We treat the base statistics from a simulated dataset as independent variables and the logarithm of the coalescence rate as a dependent variable, and perform a fit. This gives us a summary statistic (a function of the base statistics) which predicts the log coalescence rate. We similarly fit a summary statistic to predict the hybrid speciation number.

**Fig. 8.—:**
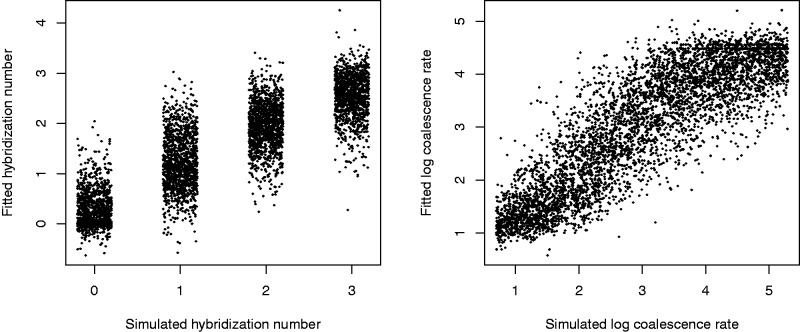
Summary statistics were derived from the base statistics to fit the log coalescence time and hybrid speciation number for simulated data for example 4. Those fits are shown here. The *x*-axis of the first plot is “jittered”, randomly shifting points from their (always integer) true values.

The summary statistics were fitted using 10,000 simulations. The fits were linear with interaction terms, and are illustrated in figure 8. As there are 11 base statistics, this gives 1 (constant) + 11 (first-order) + 55 (interaction terms) = 67 fitted coefficients. When evaluated by AIC, only 15 (log coalescence rate) or 8 (hybrid speciation number) coefficients were discarded as statistically insignificant. The standard deviations of residuals of the fits were 0.643 for log coalescence rate (i.e., coalescence rate estimate is typically within a factor of 2 of being correct) and 0.550 for the hybrid speciation number. The residuals are nearly uncorrelated (correlation coefficient 0.19).

**Fig. 9.—:**
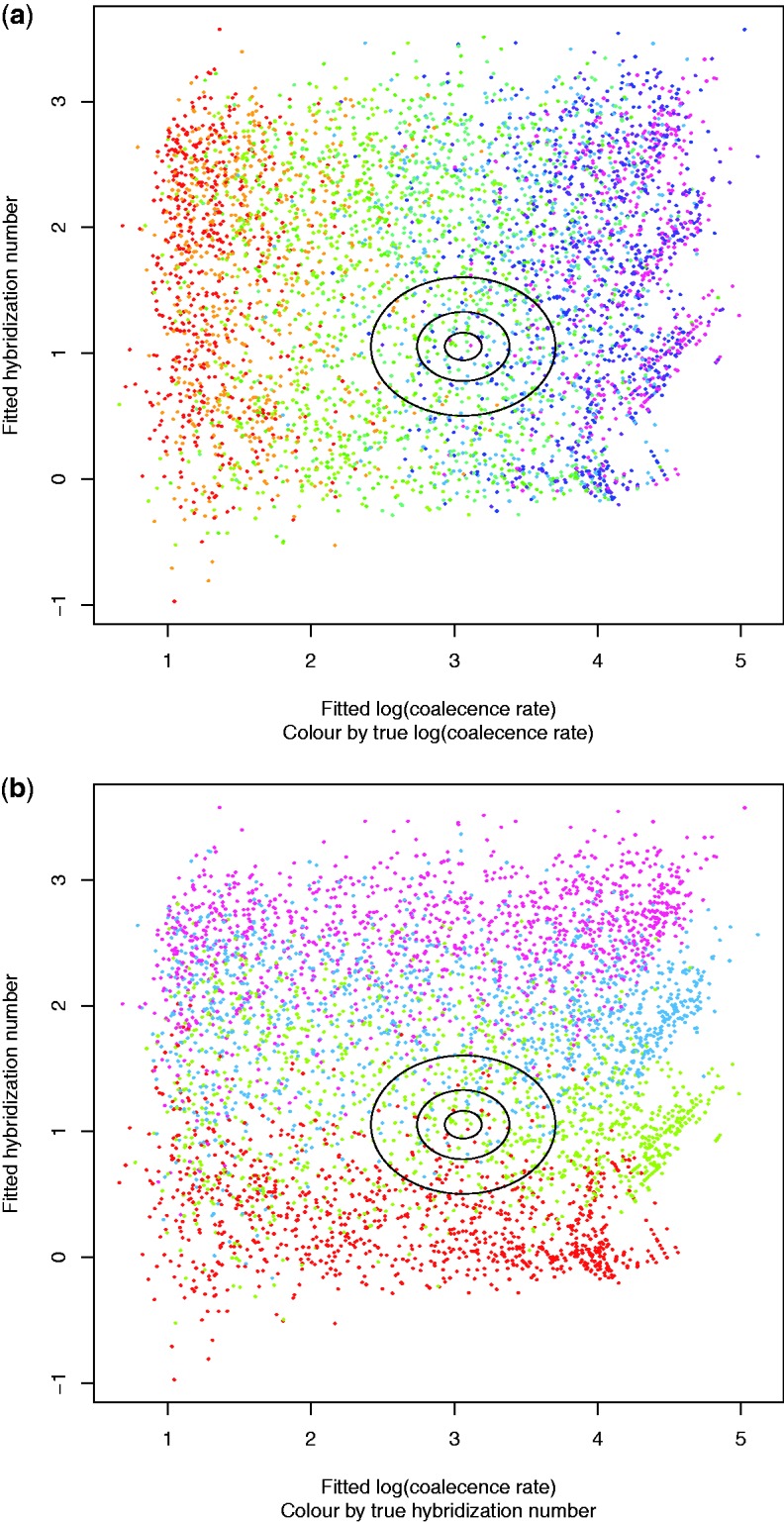
For example 4, the two summary statistics (figure 8) are plotted against each other. The two plots are identical except for color coding. The ellipses (radii 1, 0.5, and 0.2 standard deviations of the residuals) mark the summary statistics of the actual data (106 gene trees of eight yeast taxa). The ABC sample was taken from the innermost ellipse. (A random 5000-point sample is plotted here.).

**Fig. 10.—:**
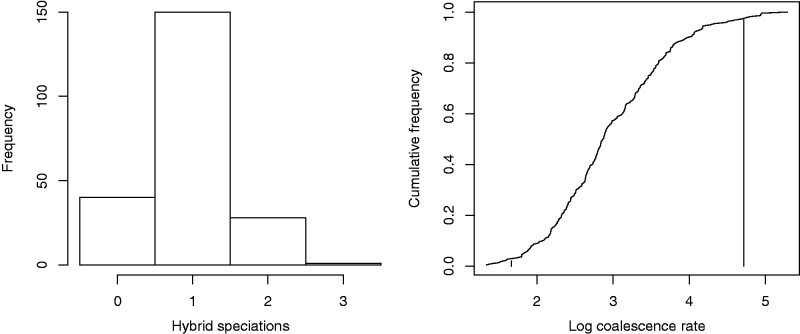
Posterior distributions of hybrid speciation number and log coalescence rate from the ABC analysis (example 4). The vertical lines mark the 95% confidence interval for the log coalescence rate. The analysis is inconclusive as to whether hybridisation has affected this phylogeny, with 18% of the sample having no hybrid speciation events.

The ABC analysis used 100,000 simulations, not overlapping with the 10,000 simulations used to fit the summary statistics. The ABC sample was taken as simulations with summary statistics within radius 0.2 (in units of standard deviation of residuals) of the summary statistics taken from the true data (table 5, fig. 9) 219 simulations were selected. Figure 10 shows the distributions of coalescence rate and hybrid speciation number in the ABC sample. 18% of the ABC sample has no hybrid speciation, so we have not got significant evidence for or against hybridization in this phylogeny. (Hybrid speciation number counts in the sample are 40, 150, 28, and 1 for 0, 1, 2, or 3 hybrid speciations, respectively.)

## Discussion

We have introduced a new flexible simulator of hybrid evolution and provided proof of concept that it could be used in an ABC framework to make inferences about different sources of incongruence. Phylogenetic methods have been developed to infer species trees in the presence of stochastic error and ILS (Maddison and Knowles 2006; Liu and Pearl 2007; Kubatko 2009; Heled and Drummond 2010), another potential use of our simulator would be to test how well these methods perform in the presence of hybridization.

There are features that we did not model at this stage. Our simulator considers homoploid hybridization, but not yet polyploidy hybridization, which is undoubtedly of much significance for some evolutionary lineages (Van de Peer et al. 2009; Jiao et al. 2011). Spatial structure is also doubtlessly important (Abbott et al. 2013), but we have not included it. We also ignore extinction, and while we allow for asymmetry we do not model selection/adaptation. We are aware that hybrid speciation and introgression can act to either enhance or restrict opportunities for speciation depending on context (Abbott et al. 2013). In our model, we assume independence between hybrid speciation events. A gene which introgressed from one lineage to another is, in a following introgression event, no more or less likely to introgress again than any other gene.

In our simulation of the coalescent, we do not account for a population bottleneck during hybrid speciation or introgression, and we also impose a single tree on each gene, thus denying the possibility of uncoalesced lineages of a single gene coming from different ancestor species via a reticulation event. Our current implementation of coalescence also has the limitation that all lineages at a given epoch have the same coalescence rate (i.e., we have effectively assumed that all species have the same population size, although we allow that universal population size to vary deterministically over time).

We suggest a suite of base statistics that can be used to summarize the information in sets of gene trees. This is an area of research that is relatively unexplored, it is likely that other researchers will think of alternative base statistics. Until a wider range of datasets have been explored it seems wise to keep an open mind about which statistics will be most useful. In any case, some of the latest approaches to ABC (Fearnhead and Prangle 2012) begin with a very wide range of base statistics and use an initial simulation step to find summary statistics as transformations of the base statistics that are most powerful for parameter inference.

We expect that for many groups of interest it will not be realistic to infer an accurate hybrid phylogeny, however, the tools provided here will be useful in inferring the extent to which hybridization has been important in comparison to other processes such as incomplete-lineage sorting.

The HybridSim software is available at https://github.com/MichaelWoodhams/HybridSim. For simulations of the size considered in this article, it is quite fast. The longest run was 110,000 trials for the yeast dataset (example 4), which took 10 min (single threaded) on an Intel i7-3770 CPU. The next most CPU intensive example (example 2) took 150 s.

In future work we plan to use it within a semi-automatic ABC framework in order to infer relative rates of hybridization for large multi-gene datasets such as produced by Salichos and Rokas (2013) and Cranston et al. (2009).

## Supplementary Material

Supplementary material is available at *Genome Biology and Evolution* online (http://www.gbe.oxfordjournals.org/).

Supplementary Data
