# Supplementary material for: Simulating and Summarizing Sources of Gene Tree Incongruence
Source: Genome Biol Evol. 2016 Mar 26;8(5):1299–315. doi: 10.1093/gbe/evw065 (PMC4898792; doi:10.1093/gbe/evw065)
Supplement: Supplementary Data [file supp_8_5_1299__index.html]

Simulating and Summarizing Sources of Gene Tree Incongruence — Supplementary Data 

# Simulating and Summarizing Sources of Gene Tree Incongruence

## Supplementary Data

files

- Supplementary Data - pdf file
- Supplementary Data - pdf file
