# Supplementary material for: Simulating and Summarizing Sources of Gene Tree Incongruence
Source: Genome Biol Evol. 2016 Mar 26;8(5):1299–315. doi: 10.1093/gbe/evw065 (PMC4898792; doi:10.1093/gbe/evw065)
Supplement: Supplementary Data [file supp_evw065_HybridSim_manual.pdf]

HybridSim Manual

Michael Woodhams

September 2015

HybridSim is a simulator for generating phylogenies in the presence of hybridization. It operates in two modes. In simple mode, it outputs a single reticulate phylogeny plus a set of trees. In statistics mode, it simulates many reticulate phylogenies and outputs a set of statistics derived from each.

Input files are in Nexus format. In simple mode, the output Nexus file includes input blocks specifying the parameters of the simulation – thus the file contains not only the output of the program, but records the exact input that produced this output, and so is fully self documenting. The output file can even be used as an input file, in which case HybridSim will throw out all of the output blocks and regenerate them. (HybridSim is "idempotent" in this mode: using the output of one run as the input to another run will produce identical output.)

HybridSim uses a lightly modified version of the PAL library version 1.5.1 [2], and BioJava version 1.8.4[3] for Nexus file parsing.

Simulation

The mathematical details behind HybridSim are described in more detail in [5], but in this section we give a brief recapitulation. HybridSim is a forwards-in-time simulator of reticulate phylogenies, allowing two types of reticulation (hybridization and introgression), with the probability of hybridization being a function of genetic distance, with rates of various processess varying with time.

Terminology

Epoch HybridSim allows some parameters to vary with time, as a series of step functions. An epoch is a period of time within which all parameters are constant. Parameters can change value only at the boundaries between epochs.

Gene Tree A gene tree represents the phylogeny of a given gene. It is derived from a lineage tree (which defines the phylogeny of the locus at which the gene sits) by a lineage sorting/coalescent random process. Nodes on the gene tree always predate the corresponding node in the lineage tree

(or network), and it may have differet topology to the lineage tree which generated it, due to incomplete lineage sorting.

Hybridization An event where a new species is formed with its initial geneome being derived from two parent species. Some proportion of its initial genome comes from one parent, the remainder from the other.

Introgression An event where a species has some proportion of its genome overwritten by that of another species. (This may be caused by hybrid individuals who then enter the gene pool of the introgressed species.) This could also be called a horizontal gene transfer event.

Lineage Tree A tree (embedded in the network) representing the lineage of a given locus. At each reticulation event, the lineage will inherit from one of the parent species only. Nodes on the lineage tree correspond exactly to speciation and hybridization events in the network.

Network A reticulate phylogeny, i.e. a phylogeny including hybridization and introgression events

Reticulation Either a hybridization or introgression (i.e. any node in the network with in-degree greater than one represents an instance of reticulation.)

Nexus File Format

HybridSim takes a Nexus file as input. This Nexus file will contain a hybridsim block and may also contain a hybridABCgrid block.

If the hybridABCgrid block is absent, HybridSim works in its simple mode of operation, each run produces a single random network. Output is a Nexus file containing the network (in extended Newick format[4]) and several sets of trees, as well as the input parameters. It can also (depending on input parametes) simulate a Dollo process which will result in a characters block. A distances block records the average genetic distance between taxa (i.e. terminal nodes in the network.)

If the hybridABCgrid block is present, HybridSim will generate many networks, and for each network a number of gene trees. It will output summary statistics for each network's set of gene trees into a (non-Nexus) text file. (In principle, a Nexus characters block could contain this information, but coaxing Biojava to produce something useful was very hard. I could revisit this, if there is demand.)

HybridSim input block

The hybridsim block contains a list of <parameter>=<value> pairs. Parameter names are not case sensitive.

Some parameters can change their value with time - they are piecewise constant functions. The "epochs" parameter specifies the times at which other parameters may change their values. It contains a parenthesis-delimited monotonically increasing list of non-negative real numbers. Time variable parameters specify a list of values, of length one greater than the length of the epochs list. For example we could have "epochs = (0.5,1)" and "speciation rate = (5,3,1)", indicating that speciation rate is 5 up to time 0.5, rate is 3 between 0.5 and 1, and rate is 1 thereafter. A single value may be specified where a list is expected, in which case that value holds for all epochs. In the above example "coalescence rate = (10)" is synonymous with "coalescence rate = (10,10,10)".

Some values are discrete distributions, which are a list of value-weight pairs. For example, "(0.1,2,0.2,1)" specifies a distribution which will evaluate to 0.1 twice as often as it evaluates to 0.2. (The distribution "(0.1,10,0.2,5)" is exactly synonymous to the above.)

Comments can be added inside square brackets, as normal for a Nexus file. These comments will not get reproduced in the output Nexus file.

coalesce (Boolean.) If true, include in the output a trees block of gene trees derived from lineage trees by coalescence. *Default = true.*

coalescence rate (List of non-negative reals.) The epoch-dependent coalescence rate. This is the rate at which two gene lineages which are able to coalesce will do so. This and "coalescence time" are different ways of setting the same variable - don't use both. *Default = (1).*

coalescence time (List of non-negative reals.) The epoch-dependent coalescence time. This is the mean time for two gene lineages which are able to coalesce will do so. This and "coalescence rate" are different (inverse) ways of setting the same variable - don't use both. *Default = (1).*

dollo rate (Real.) The rate at which Dollo characters undergo 1->0 mutations. Has no effect if "dollo sites per tree" is zero. (Dollo rate cannot vary with epoch, like e.g. speciation rate. This may change in subsequent versions of HybridSim.) *Default = 1.*

dollo sites per tree (Integer.) If non-zero, a Dollo process presence/absence data will be simulated on the network. The output will include a CHARACTERS block with "dollo sites per tree"×"number random trees" binary characters. *Default = 0.*

epochs (List of non-negative reals.) A list of zero or more times at which various other parameters can change values. Values must be in ascending order. This is the only parameter allowed to take an empty list as its value. *Default = ().*

halt reticulations (Integer.) The maximum number of reticulation events that the network can hold. The simulation will halt and produce output immediately prior to an event which would otherwise exceed this limit. *Default = 20.*

halt taxa (Integer.) The maximum number of taxa (leaf nodes) that the network can grow to. The simulation will halt and produce output immediately prior to an event which would otherwise exceed this limit. *Default = 20.*

halt time (Real.) The maximum time the simulation will run for. The simulation will halt and produce output at this time, unless the "halt taxa" or "halt hybrid" halting conditions are met first. *Default = 10.*

hybridization distribution (List of values/weights.) A discrete distribution specifying the gene genetic contribution of the first parent to a new hybrid species. *Default = (0.5,1), i.e. always 50-50 contribution of genes from each parent.*

hybridization leaf function One of "const","linear", "quadratic". Determines the rate over the entire network of hybridization events being attempted. The default "quadratic" gives the chance of a given pair of taxa attempting to hybridize being independent of the number of taxa. There is little reason to change this.

hybridization rate (List of non-negative reals.) The epoch-dependent rates at which pairs of taxa will attempt to hybridize. *Default = (1).*

introgression distribution (List of values/weights.) A discrete distribution specifying the gene genetic contribution from the introgressor species in an introgression event. *Default = (0.1,1), i.e. always 10% contribution of genes from introgressor.*

introgression leaf function As "hybridization leaf function" but for attempts to introgress. There is little reason to change this.

introgression rate (List of non-negative reals.) The epoch-dependent rates at which pairs of taxa will attempt to introgress. *Default = (1).*

number random trees (Integer.) The number of random (lineage or gene) trees to generate in the output, in a TREES block. Also affects the number of Dollo sites output (if any.) *Default = 1.*

minimum reticulations (Integer.) Should the random network be generated with fewer than this number of reticulation events, the network will be discarded and a new one generated. *Default = 0.*

reduce reticulations to (Integer.) Should this value be positive and the random network have more than this number of reticulation events, then reticulation events will randomly be removed until this number is reached. *Default = -1.*

reticulation function one of "linear", "quadratic", "step", "exponential", "snowball". See [5] for details.

reticulation threshold (List of non-negative reals.) The epoch-dependent threshold genetic difference beyond which hybridization and introgression are difficult or impossible. Interpretation depends on “reticulation function”. *Default = (1).*

seed (Long integer.) Seed to the random number generator. *Default = 4.*

speciation leaf function One of “const”,“linear”, “quadratic”. Determines the rate over the entire network of speciation events ocurring. The default “linear” gives a Yule process. There is little reason to change this.

speciation rate (List of non-negative reals.) Epoch dependent list of the rate at which a taxon will produce speciation events. *Default = (1).*

Example

```
1  #NEXUS
2  begin hybridsim;
3      epochs = (1);
4      speciation rate = (3,0.5);
5      hybridization rate = (0,2);
6      introgression rate = 0;
7      hybridization distribution = (0.1,1,0.25,1,0.5,2);
8      reticulation threshold = 1;
9      reticulation function = linear;
10     minimum reticulations = 2;
11     reduce reticulations to = 2;
12     coalesce = true;
13     coalescence rate = 6;
14     halt time = 20;
15     halt taxa = 20;
16     halt reticulations = 30;
17     dollo sites per tree = 0;
18     filo sites per tree = 0;
19     number random trees = 250;
20     [Comments can be added but will not be transfered to the output file.]
21 end;
```

In this example, we have two epochs, first from $t = 0$ to $t = 1$, then for $t > 1$ (line 3). Speciation occurs at rate 3 during the first epoch and rate 0.5 in the second epoch (line 4). Hybridization does not occur (rate 0) in the first epoch and has rate 2 (per pair of species) in the second (line 5). Introgression never occurs (line 6). New hybrids get 50-50 gene contributions from their parents 50% of the time, 75-25 contribution 25% of the time and 10-90 25% of the time (line 7). As introgression rate is zero, the “introgression distribution” is immaterial and we have not specified it. The chance of an attempted reticulation succeeding linearly decreases (line 9) from 100% at genetic distance of zero, to

0% at genetic distance of 1 (line 8). The generated network will have exactly two reticulation events (lines 10 and 11.) 250 gene trees (line 19) will be generated by a coalescent process (line 12), with coalescence rate 6 (line 13) at all epochs. The generated network will have 20 leaf taxa (line 15) (unless the halt time of 20 (14) or maximum reticulations (prior to being reduced) of 30 (line 16) occur first, which on these parameters is unlikely.) No Dollo data will be produced (line 17, could be omitted as this is the default.)

HybridABCGrid Nexus Block

This block is optional. It allows running HybridSim multiple times with varying parameters, and recording summary statistics from each run. In this mode, the output file is a space-delimited table with columns for the parameters iterated over and for summary statistics on the generated trees.

Like the HybridSim block, it consists of <parameter> = <value> pairs. Any valid parameter for HybridSim can be a HybridABCGrid parameter. (In which case, the value specified in the hybridsim block will be ignored, as the hybridabcgrid block value takes precidence.) The "value" specifies a column name and set of values to iterate over for that parameter. In one form we use "{}" and a list of values separated by "|"

```
<parameter> = <columnName>{<value1 >|<value2 >...}
```

e.g.

```
epoch = EPOCH{(1,2)|(2,3)}
coalesence rate = cRate{5|15}
```

The alternative form specifies a column name followed by a specification of numbers to iterate over, enclosed in parentheses "()". The number iteration specification consists of a start value, stop value and optional step size value, separated by ":". E.g.

```
reduce reticulations to = HYBR(0:5)
coalescence rate = cRate(5:15:10)
```

In either case, the column name is used as the header to the column for this value in the output text file. If you want multiple simulations on a given set of parameters, iterate over seed:

```
seed = SEED(1:500)
```

There are additional parameters to the HybridABCGrid block to control the summary statistics which are output. Both take a list of integers. The lists are surrounded by "{}" and values separated by "|".

split incompatibility thresholds (List of integers.) Gives the numbers ("#") in the "SI-#" statistics [5]. *Default = {1|2}, i.e. output will have SI-1 and SI-2 statistics.*

rare splits thresholds (List of integers.) Gives the numbers ("#") in the "RS#" statistics[5]. *Default = {1}.*

(It is also possible to define new statistics which are polynomials in the existing statistics, but I choose not to document that feature. In the unlikely event someone cares, they can read the source code.)

Filo integration

Filo[1] is a very general sequence simulation program, able to simulate sequences from inhomogeneous models (different DNA models on different branches) and reticulate phylogenies (through different trees applying to different parts of the sequence.) Filo uses Nexus format for input. HybridSim is able to generate a useable Filo input block in its output, so HybridSim's output file can be used as an input file to Filo. To do this, it is necessary to set "filo sites per tree" to a non-zero value, and to provide a template Filo block in the HybridSim input file. The input Filo block passes through to the output, with the following edits:

- Any 'tree' and 'treeparams' fields in the input are ignored. New 'tree' and 'treeparams' fields are generated for the output, the trees being random lineage trees.
- The params field will be modified to give sequence length equal to "filo sites per tree" × "number random trees".
- If the input contains a 'run' command it will be ignored. A new 'run' command will be added at the end of the block.

This does not exercise the full range of Filo's capabilities, for example inhomogeneous models are not supported by this mechanism. For example, with Filo template block

```
begin filo;
        output
                format = fasta, nexus, raw
                filename = hybridoutput
                precision 5
        ;
        matrix HKY = HKY85 0.2 0.5 [ 0.25 0.05 0.25 0.45 ];
        params
                l 400
                indel 0
        ;
        run;
end;
```

we could end up with a Filo block in the output which looks like

```
BEGIN Filo;
        output
                format = fasta, nexus, raw
```

```
                filename = hybridoutput
                precision 5
                ;
        matrix HKY = HKY85 0.2 0.5 [ 0.25 0.05 0.25 0.45 ];
        params
                l 40
                indel 0
        ;
        tree t1 =((C:0.6028567,F:0.6028567):0.8836763,(((D:0.4711576,H:0.471157
        treeparams t1
                l 20
        ;
        tree t2 =((A:1.3671101,((G:0.4427599,B:0.4427599):0.3076275,(H:0.390438
        treeparams t2
                l 20
        ;
        run ;
END;
```

EBNF

The HybridSim block:

```
HybridSim block = "begin hybridsim", {hybridsim assignment | nexus comment}, "en
nexus comment = "[", text, "]" ;
hybridsim assignment = epoch assignment | list assignment | discrete distributio
          integer assignment | hybrid function assignment | boolean assignment |
epoch assignment = "epochs", "=", real list, ";" ;
real list = "()" | nonempty real list ;
nonempty real list = nonnegative real number | "(", list of reals, ")" ;
list of reals = nonnegative real number, {",", nonnegative real number} ;
list assignment = list parameter, "=", nonempty real list, ";" ;
list parameter = "speciation rate" | "hybridization rate" | "introgression rate"
              "reticulation threshold" | "coalescence rate" | "coalescence time"
discrete distribution assignment = distribution parameter, "=", distribution lis
distribution parameter = "hybridization distribution" | "introgression distribut
distribution list = "(", zero to one real, ",", nonnegative real number, {",", z
zero to one real = [0], ".", digit, {digit}
integer assignment = integer parameter, "=", nonnegative integer, ";" ;
integer parameter = "halt taxa" | "halt hybrid" | "number random trees" | "dollo
long integer assignment = long integer parameter, "=", [-], nonnegative integer,
long integer parameter = "seed" ;
hybrid function assignment = reticulation function parameter, "=", reticulation
reticulation function parameter = "reticulation function" ;
reticulation function = "linear" | "quadratic" | "step" | "exponential" | "snowb
```

```
leaf function assignment = leaf function parameter, "=", leaf function, ";" ;
leaf function paramter = "speciation leaf function" | "hybridization leaf functi
leaf function = ("const" | "linear" | "quadratic") ;
boolean assignment = boolean parameter, "=", ("true" | "false"), ";" ;
boolean parameter = "coalesce" ;
real assignment = real parameter, "=", nonnegative real number, ";" ;
real parameter = "dollo rate" | "halt time" ;

nonnegative real number
digit
nonnegative integer
text
```

Comments:

Any “nonempty real list” must either be of length 1, or length one greater than the “epochs” list. The “epochs” list must be in increasing order. “zero to one real” can also include exponential form (e.g. “1.4e-3”) so long as it lies between zero and one. Nexus comments can be nested - I haven’t attempted to represent this in the grammar.

All input is not case sensitive. Within a parameter name, white space is significant (e.g. “halt time” must have a single space, not two spaces or a tab character.) Outside of parameter names it is not significant, except there cannot be a new line character between a parameter name and the following “=”.

The HybridABCGrid block:

```
HybridABCGrid block = "begin hybridabcgrid", {hybridgrid assignment | nexus comm
hybridgrid assignment = iterator assignment | summary stats specifier;
summary stats specifier = ("split incompatibility thresholds" | "rare splits thr
iterator assignment = hybridsim parameter, "=", column label, iterator, ";", ;
iterator = numeric iterator | list iterator;
numeric iterator = "(", positive integer, ":", positive integer, [":", positive
list iterator = "{", text, { "|", text }, "}" ;

hybridsim paramter = list parameter | distribution paramter | integer parameter
positive integer
```

Comments:

In a “list iterator”, the text must be a legal value for the corresponding HybridSim parameter.

Command Line Options

The options are:

-i <inputfile> The name of the input file. *Default = input.nex*

-o <outputfile> The name of the output file. *Default = output.nex or output.table*

-s <number> Sets the pseudorandom number generator seed. Overrides any value set in hybridsim Nexus block, but not in HybridABCGrid block. (The output Nexus file will record this number.)

The space between flag name and argument is optional. For example

```
java -jar hybridsim.jar -i example.nex -o output5.nex -s5
```

Nexus Output Format

If there is no HybridABCGrid block, the output will be a Nexus file. This file is useable as input to HybridSeq, and if so used will recreate itself exactly. There are three output trees blocks. These are identified with comments. The output nexus file will contain blocks:

HybridSeq containing the input parameters to the simulation

Taxa containing the taxon names for the network, which will be "A", "B", etc.

Trees The first trees block will exhaustively list the lineage trees with their probabilities specified in comments. Tree names start with "ELT" (for "exact lineage tree".) This block may be omitted if the number of lineage trees is too large. (The number grows exponentially with the number of reticulation events.)

Trees The second trees block contains randomly selected (with weight equal to their probability) lineage trees. The number of randomly selected trees is determined by the "number random trees" parameter. These trees have names starting "RLT" (random lineage tree.)

Trees The third trees block contains coalescent (i.e. gene) trees derived from the lineage trees of the previous block. These trees have names starting "CT" (coalescent tree.) This block will be absent if parameter "coalesce" is false.

eNewick This block specifies the network in 'extended Newick' format.[4]

Distances This block records the average distance between taxa. If "reduce reticulations to" has non-default value, the Distances block is not output. (This is because eliminating reticulations modifies the distance matrix and there currently is no code to recalculate it correctly when this happens. Such code could be added if there is a need for it.)

References

[1] Michael Charleston. Filo, 2009.

[2] Alexei Drummond and Korbinian Strimmer. PAL: an object-oriented programming library for molecular evolution and phylogenetics. *Bioinformatics*, 17(7):662–663, 2001.

[3] R. C. G. Holland, T. A. Down, M. Pocock, A. Prlić, D. Huen, K. James, S. Foisy, A. Dräger, A. Yates, M. Heuer, and M. J. Schreiber. Biojava: an open-source framework for bioinformatics. *Bioinformatics*, 24(18):2096–2097, 2008.

[4] M. M. Morin and B. M. E. Moret. NetGen: generating phylogenetic networks with diploid hybrids. *Bioinformatics*, 22(15):1921–1923, 2006.

[5] Michael D. Woodhams, Peter J Lockhart, and Barbara R. Holland. Modeling hybrid evolution. *In preparation.*
