# Supplementary material for: Simulating and Summarizing Sources of Gene Tree Incongruence
Source: Genome Biol Evol. 2016 Mar 26;8(5):1299–315. doi: 10.1093/gbe/evw065 (PMC4898792; doi:10.1093/gbe/evw065)
Supplement: Supplementary Data [file supp_evw065_supplementary.pdf]

Simulating and summarizing sources of gene tree incongruence — Supplementary material

Michael D. Woodhams, Peter. J. Lockhart and Barbara R. Holland

March 9, 2016

Consensus networks

We show Splitstree [Huson and Bryant, 2006] consensus networks [Holland et al., 2004] for some random tree sets from example two. Example two presented a sequence of models, starting with no hybridisation and large effects of lineage sorting (figure S1) and ending with frequent hybridisation but very small lineage sorting effects (figure S4). The networks generally get progressively less tree-like as hybridisation increases, but with a lot of random variation.

The networks presented are simply for the first three simulations for each set of parameters, and so constitute a (small) random sample.

References

Barbara R Holland, Katharina T Huber, Vincent Moulton, and Peter J Lockhart. Using consensus networks to visualize contradictory evidence for species phylogeny. *Molecular Biology and Evolution*, 21(7):1459–1461, 2004.

D. H. Huson and D. Bryant. Application of phylogenetic networks in evolutionary studies. *Mol. Biol. Evol.*, 23(2):254–267, 2006. doi: 10.1093/molbev/msj030.

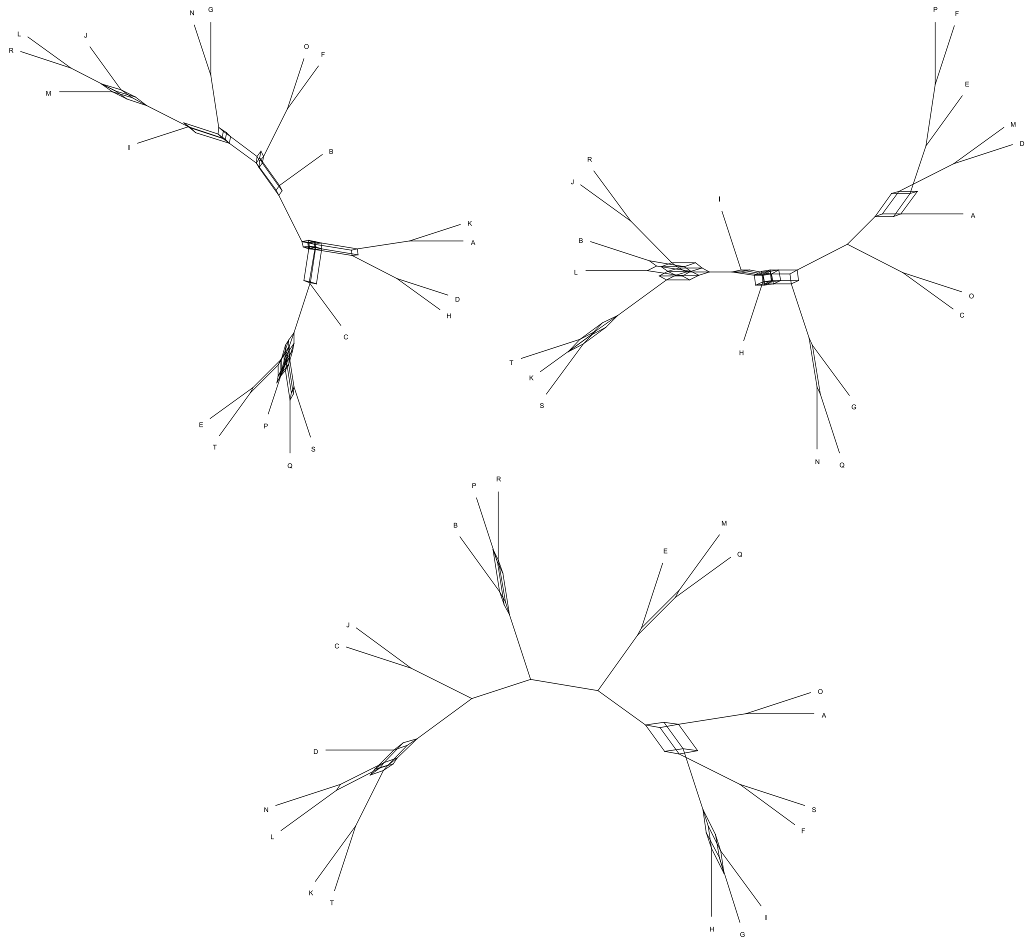

Figure S1: Gene trees generated with hybridisation rate zero and coalescence rate 6. Non-treeness is caused by lineage sorting, and is small scale and localized.

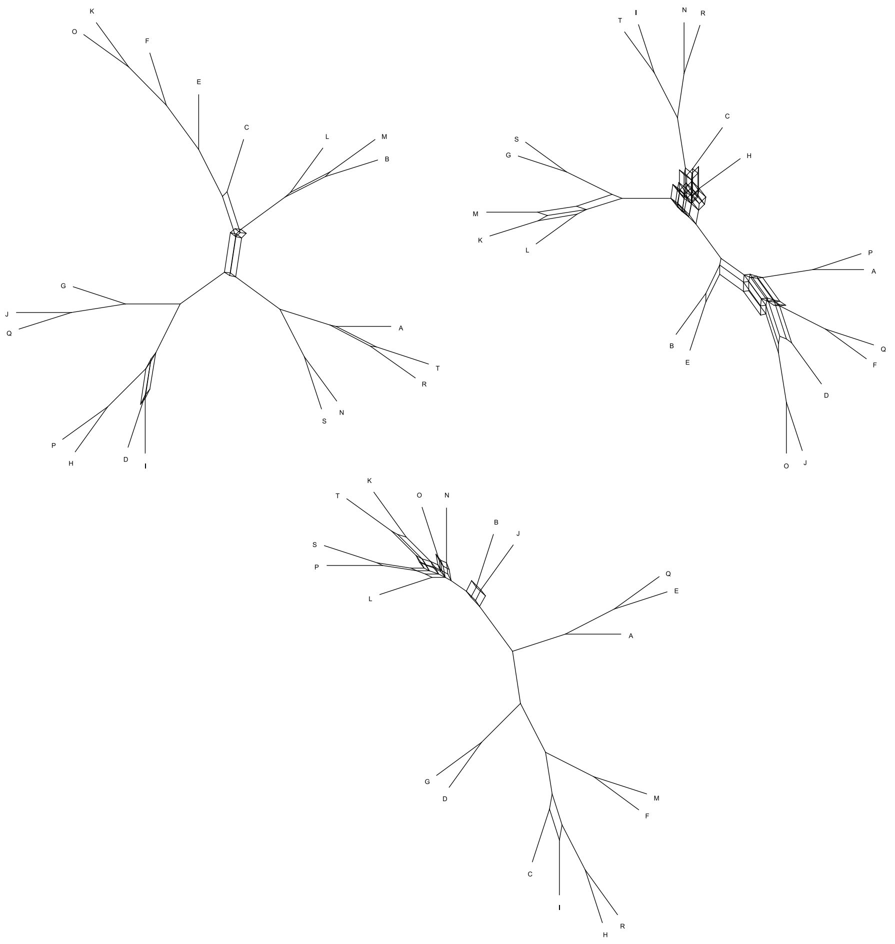

Figure S2: Gene trees generated with hybridisation rate 0.1 and coalescence rate 10. Compared to the previous figure, lineage sorting is less important (due to a higher coalescence rate) and hybridisation is now a factor. The non-treeness of the networks has increased and become less localized.

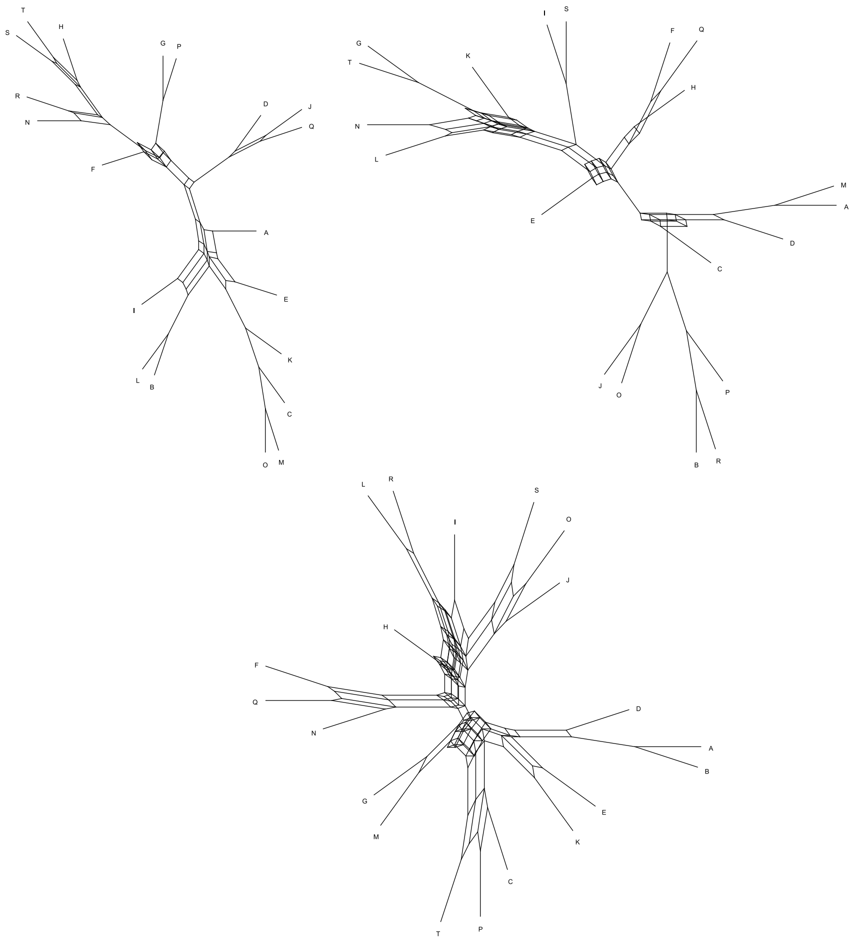

Figure S3: Gene trees generated with hybridisation rate 0.26 and coalescence rate 30. This continues the sequence towards less lineage sorting and more hybridisation. Non-treeness now can be extensive and global (e.g. the third network.)

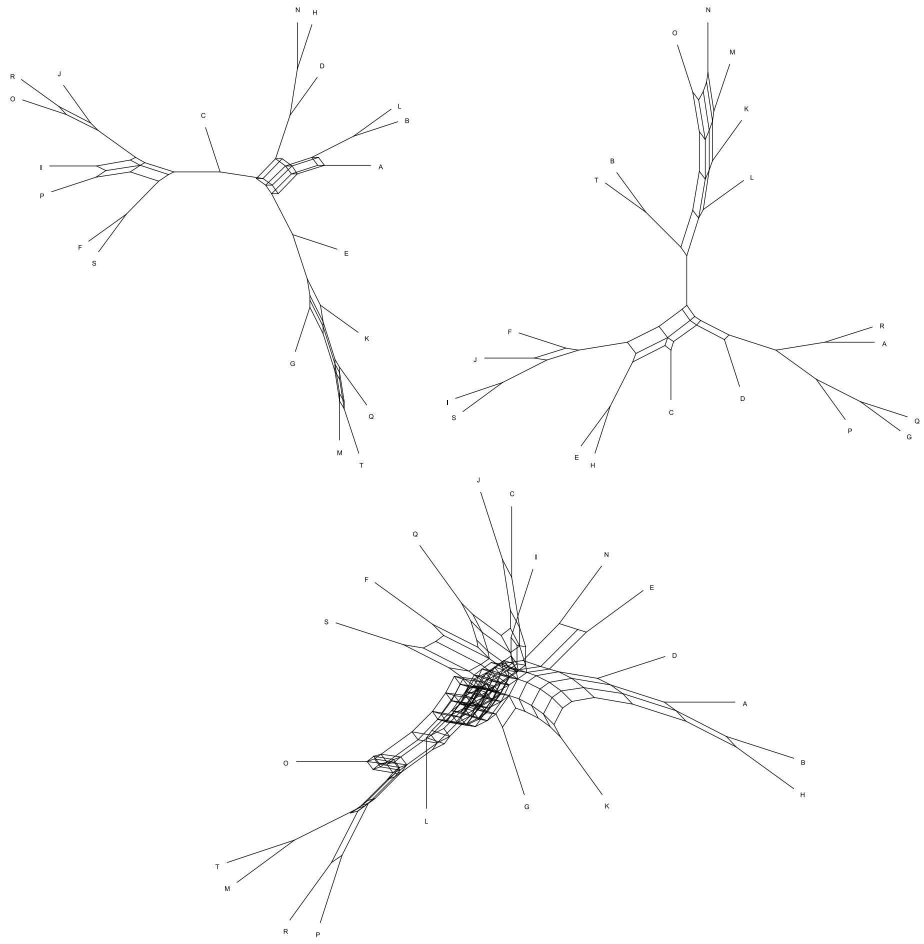

Figure S4: Gene trees generated with hybridisation rate 0.4 and coalescence rate 1000. With such a high coalescence rate, lineage sorting effects are essentially removed. There is little to distinguish these networks from the hybridisation 0.26, coalescence 30 networks of the previous figure.
